# Conformational Changes of Congenital FVII Variants with Defective Binding to Tissue Factor ARG304GLN (FVII Padua), ARG 304TRP (FVII Nagoya) and ARG79GLN (FVII Shinjo or Tondabayashi)

**Published:** 2013-12

**Authors:** Andrea Cristiani, Silvia Vettore, Luisa Sambado, Alessandro Bulfone, Stefano Moro, Antonio Girolami

**Affiliations:** 1 Department of Medicine, University of Padua Medical School, Padua, Italy;; 2 Department of Pharmaceutical and Pharmacological Sciences, Padua, Italy;; 3 CRS4 - Biomedicine Sector, Cagliari, Italy

**Keywords:** Factor VII deficiency, molecular conformation, Tissue Factor and Thromboplastins

## Abstract

**Background:**

The relation between Factor VII (FVII) and tissue thromboplastin is not completely clarified, yet. Three FVII abnormalities, FVII Padua (Arg304Gln), FVII Nagoya (Arg304Trp) and FVII Shinjo or Tondabayshi (Arg79Gln) show different FVII activity according to the tissue Tissue Factor (TF) used in the assay system (rabbit brain, human placenta or human recombinant and ox brain).

**Objectives:**

To investigate the possible existence of common conformational changes with regard to different tissue factors in these three FVII variants.

**Material and methods:**

Crystal structure analysis and “visual inspection” of FVII were deeply performed to select a crystallographic template for the in silico mutagenesis procedure of FVII Arg79Gln, Arg304Gln and Arg304Trp.100ns 300K NVT large-scale molecular dynamics simulation on GPU were applied to the models of FVII. The aims of this run was to describe at molecular level the influence of the mutation on the protein structure and function.

**Results:**

The molecular modelling of those three variants has shown common features in spite of the different location of the mutation involved (the first epidermal growth factor for the Arg79Gln and the catalytic region for the Arg304Gln or Arg304Trp). Molecular dynamics studies have shown in fact that the mutant FVII, shows a decreased flexibility or freezing of the protein conformation of FVIIa with regard to TF. This results in the formation of a defective FVIIa-TF complex that justifies the different clotting results observed in these variants according to the TF used.

**Conclusions:**

The conformational studies may supply useful information on the structure- function relation of clotting factors.

## INTRODUCTION

Congenital Factor VII (FVII) deficiency is characterized by a variable bleeding tendency and, from a laboratory stand point, by a prolonged Prothrombin time (PT) and a normal Partial thromboplastin time (PTT) and Thrombin time (TT) (1-4). Usually FVII level in homozygotes or compound heterozygotes is less than 10% of normal whereas it is around 50% of normal in heterozygotes. Two forms of FVII deficiency are known, namely type 1 and type 2.

In the first case, FVII activity and antigen are equally decreased giving origin to cases of “true” deficiency or cross reacting material (CRM) negative. In the second, there is a discrepancy between FVII activity and FVII antigen in the sense that the latter is always higher than the activity counterpart and sometimes it is actually normal (4).

The purpose of the present study was to compare the molecular models of the three variants known to have, at the homozygote level, a sharp discrepancy between FVII activities obtained using thromboplastins of different origin in the assay system. The three variants are FVII Padua (Arg304Gln), FVII Nagoya (Arg304Trp) and FVII Shinjo or Tondabayashi (Arg79Gln) (5-10).

Little is known about the conformational changes occurring in the FVII variants during the binding process with Tissue Factor (TF) (11-14).

## MATERIALS AND METHODS

### Coagulation tests

Data pertaining to FVII Padua patients were gathered from personal files, and previous publications on the subject (5, 15, 16).

The clotting, genetic and clinical data pertaining to FVII Nagoya (Arg304Trp) and to FVII Shinjo or FVII Tondabayashi (Arg79Gln) were gathered from the already published papers (8-10).

The main clotting and clinical features of the three defects investigated are gathered in Table 1.

**Table 1:** Main features of the three FVII defects investigated

Defect	FVII activity (% of normal)				FVII Antigen (% of normal)	Bleeding tendency	Mutation (Exon)	Comments	Authors (year)
	Ra	H	Re	Ox					

FVII Padua	7	30	22	100	100	Mild or none	Arg304Gln (8)	Six homozygotes	Girolami *et al* (1970-1985)
FVII Nagoya	5	/	16	60	100	none	Arg304Trp (8)	One homozygote	Matsushita *et al* (1994)
FVII Shinjo	1	100	/	150	115	none	Arg79Gln (4)	One homozygote	Takamiya *et al* (1995)
FVII Tondabayashi	7	40	/	60	65	none	Arg79Gln (4)	One homozygote	Takamiya *et al* (1998)

Ra, Rabbit brain; H, Human tissue (human placenta); Re, Human recombinant; Ox, Ox brain.

### Modelling section studies

**Protein Data Bank screening, visual inspection, in-silico mutagenesis.** The Protein Data Bank (PDB) (17) contains 37 crystal structures of FVII and 62 of tissue factor (TF). Based on the criteria of sequence crystallization completeness, high resolution, human species, wild type(WT) and apo-form we selected 1J9C (18) PDB entry as FVIIa: TF starting complex for performing in-silico investigations of the molecular effect of Arg79Gln, Arg304Gln and Arg304Trp mutations. Based on the selection criteria previously described, we selected a template for FVII zymogen resulting in 1JBU (19). 1JBU is the only one wt public reference structure of the not-activated form of FVII. Part of the protease domain was disordered; thus, we reconstructed loops Leu213-Glu215 and Gly285-Ala292 with homology modelling and AMBER FF99 (20, 21) parameterization.

We aligned with MOE 2010.10 (22) and with CLC Viewer 6 (23) the sequence of the TF contained in 1J9C PDB entry with the sequences of human (Uniprot: P13726) (24), rabbit (Uniprot: P24055) (25) and bovine (Uniprot: P30931) (26) TF.

**Figure 1:**
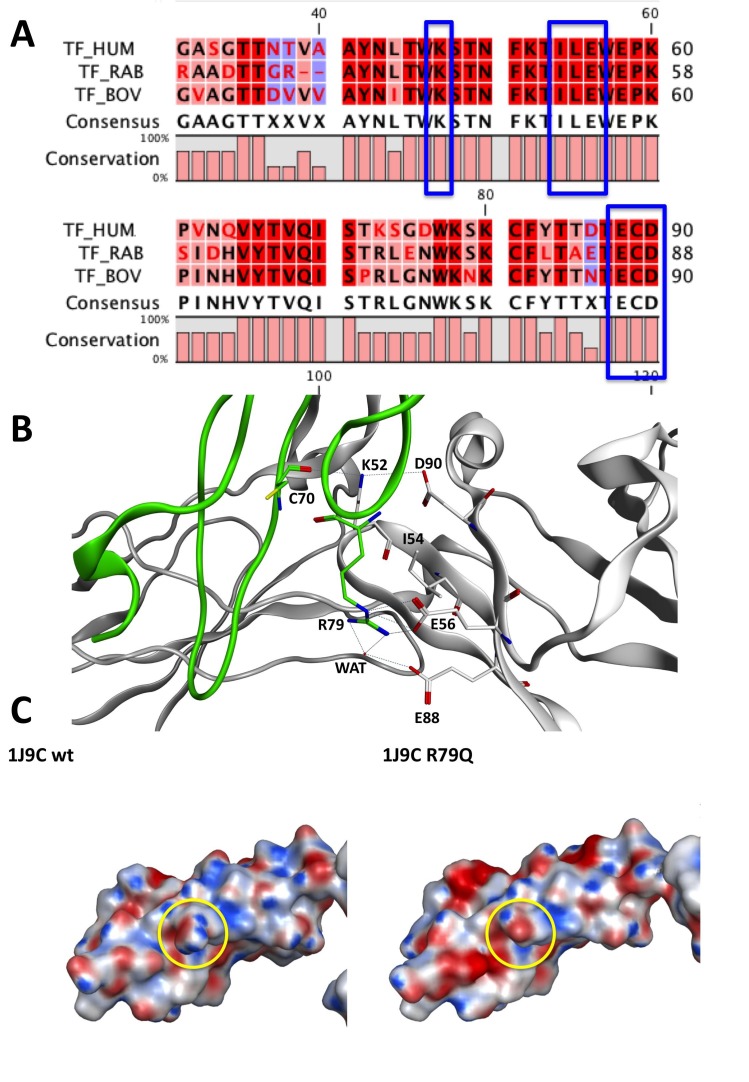
FVIIa and T.F. complex of different species (Rabbit, human, bovine) investigated. A, The picture shows a part of the MSA of human, rabbit and bovine tissue factor. The blue boxes evidence the polar amino acids interacting with R79 located in the L-chain of FVIIa in 1J9C crystal structure. These amino acids are totally conserved among the species in analysis; B, The image illustrates the polar interactions between the L-Chain of FVIIa and the TF in 1J9C crystal structure. The L-chain is represented as a green ribbon while the TF is shown in white cartoon; C, The picture shows the electrostatic surfaces of a part of the L-chains of 1J9C WT and 1J9C Arg79Gln mutant. The yellow circles underline the differences in terms of electrostatic potentials in the Arg79 mutation area. Hum, Human; Bov, Bovine; Rab, Rabbit.

We selected within a radius of 4.5 Å in the multiple sequence alignment (MSA) the amino acid interacting with Arg79 that belong to the L-chain of FVIIa; Ile54, Glu56, Glu88, Asp90 of the human TF are conserved in the rabbit and in the bovine TF sequences (Fig. 1A).

The molecular modelling suite MOE 2010.10 was used to visual inspect the crystal structures and to generate the in-silico mutants, which have been parameterized with Amber FF99SB force-field and submitted to 3 cycle of conjugated gradients minimizations in order to geometrically optimize: the side chain of the mutants, the 4.5 Å molecular environment around the mutation and the entire FVIIa:TF complex. Positional restrains have been applied on α-carbons. We applied the procedure to generate in-silico Arg304Gln and Arg304Trp mutants and their optimization to FVII “Zymogen”, too.

**Large-scale molecular dynamics.** The FVIIa:TF and FVII “Zymogen” models have been submitted to ClickMD [27] GPU-based MD pipeline and solvated with TIP3P waters in a triclinic box of these dimensions: X=112.73Å, Y=89.10Å, Z=105.26Å. for 100 ns NVT 300K. Before large-scale MD runs the molecular systems have been minimized for 100000 steps with conjugated gradient methods and equilibrated for 1 ns with αC-positional restrains in order to keep the protein conformation. ACEMD 2325 (28) was used as MD engine on nVidia GTX580 with 4 fs time step for production. NAMD 2.8 (29) was used as MD engine for minimization and equilibration phase.

### Analysis

The analysis of the trajectory produced by ClickMD has been obtained with VMD-1.9 (30), RMSD Trajectory Tools 2.01 (31) and RainbowRMSD (27).

## RESULTS

### Coagulation Studies and Clinical Status

The most striking aspect of the three defects is the variable level of FVII activity level obtained according to the thromboplastin used in the assay system (Table 1). There are minor differences in FVII levels, but the pattern is constant. Ox brain thromboplastins yield always normal FVII activity, whereas rabbit brain reagents are associated with low levels. Thromboplastin of human origin gives intermediate levels of activity. Almost all patients are asymptomatic (5-10). Patients with FVII Padua present, sometimes, a mild bleeding tendency (5, 6). The clinical picture reflects, in other words, the normal FVII activity level observed with ox brain thromboplastin and not the low level obtained with rabbit brain thromboplastins.

### Modelling Section

**Crystal structure Analysis.** Analyzing the 1J9C crystal structure, it is evident that Glu56 makes a salt-bridge with the side chain of Arg79 while Glu88 makes two hydrogen bonds with the guanidine group of Arg79; one of the two last H-bonds is mediated by the presence of a water molecule (Fig. 1B). Asp90 of human TF interacts with Lys52, which is conserved on rabbit and bovine TF (Fig. 1B), and interacts with Cys70 on the human L-chain of FVIIa (Fig. 1B).

Coupling the multiple sequence alignment (MSA) information to the analysis of 1J9C crystal structure we can suggest that the inspected interactions between human TF and the L-chain of FVIIa are probably conserved among rabbit and bovine species. Thus, it is allowed supposing that Arg79Gln mutation within the L-chain of FVIIa alters the polar interactions between rabbit TF and FVIIa and between bovine TF and FVIIa.

More in depth, we supported our hypothesis on the interactions of the rabbit TF by superimposing the human TF sequence of 1J9C with the rabbit TF sequence contained in the crystal structure of 1A21 (32) PDB entry.

We visually inspected the region of interactions between FVIIa and TF depicted in 1J9C crystal structures 4.5 Å around the mutagenesis site of Arg79. Arg79 interacts with Glu24 and Glu56 of TF, which are conserved in rabbit (P24055) and bovine (P30931) species. We performed the same analysis for Arg304 mutagenesis site evidencing differences in the sequence of the Ser71-Ser74 β-turn and the particularly in the Ala112-Glu123 loop.

1JBU contains the crystal structure of FVII “Zymogen” and it is the only one public reference structure of the not activated form of FVII.

The superimposition of the FVII H-chain (PDB: 1JBU) and FVIIa H-chain (PDB: 1J9C) shows that the loop Val302-Met327 containing the 304 mutation site and interacting with TF undergoes to major conformational changes upon the TF binding and activation.

**Figure 2:**
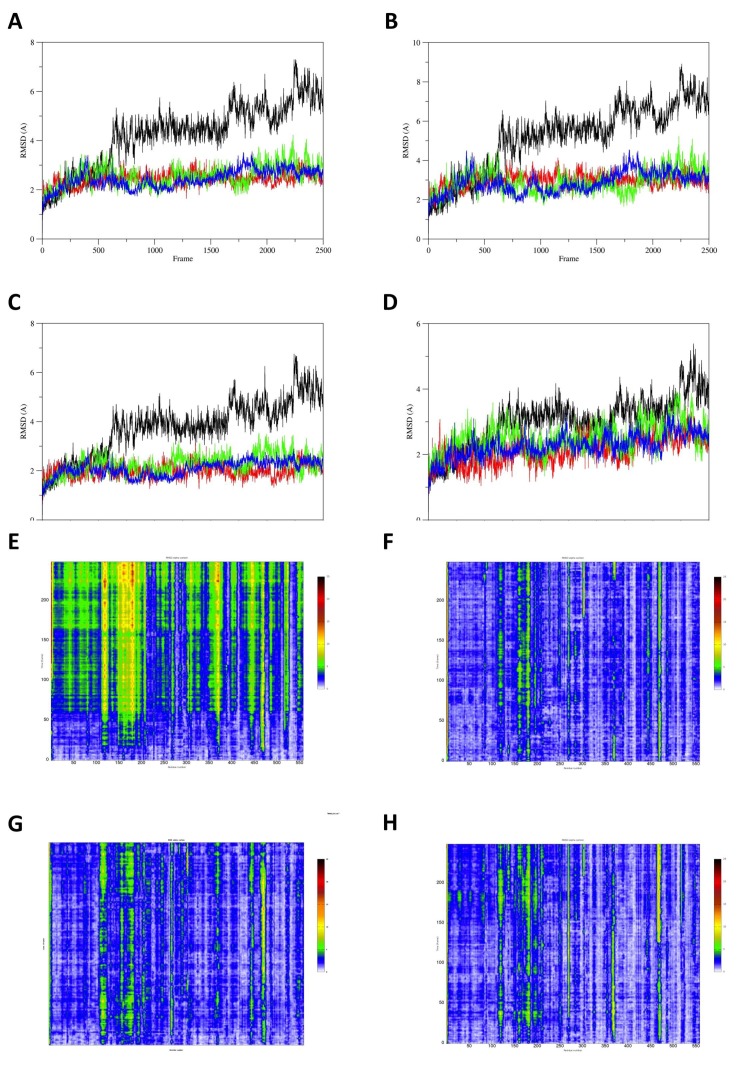
Comparison of FVIIa WT with the three mutations investigated. The discrepancy between WT and the three mutations are always present but are more evident in panels A, B and C. A, The graph shows the comparison of the protein RMSD over 100 ns NVT MD simulation of the human FVIIa:TF complex based on 1J9C crystal structure. The WT model is depicted in black while the Arg304Gln, the Arg304Trp and Arg79Gln mutants are respectively colored in red, green and blue; B, The graph shows the comparison of the protein RMSD over 100 ns NVT MD simulation of the human TF based on 1J9C crystal structure. Colors have the same meaning as in panel A; C, The graph shows the comparison of the protein RMSD over 100 ns NVT MD simulation of the human FVIIa H-chain based on 1J9C crystal structure. Colors have the same meaning as in panel A; D, The graph shows the comparison of the protein RMSD over 100 ns NVT MD simulation of the human FVIIa L-chain based on 1J9C crystal structure. Colors have the same meaning as in panel A; E, The graph represents the RainbowRMSD over 100 ns NVT 300K molecular dynamics simulation of the human 1J9C FVIIa:TF WT complex; F, The graph represents the RainbowRMSD over 100 ns NVT 300K molecular dynamics simulation of the human 1J9C FVIIa:TF Arg304Gln complex; G, The graph represents the RainbowRMSD over 100 ns NVT 300K molecular dynamics simulation of the human 1J9C FVIIa:TF Arg304Trp complex; H, The graph represents the RainbowRMSD over 100 ns NVT 300K molecular dynamics simulation of the human FVIIa:TF Arg79Gln complex.

**Molecular dynamics results.** The root mean-square deviation (RMSD) analysis of the the FVIIa:TF WT, Arg304Gln, Arg304Trp and Arg79Gln suggests a role of the mutations in stabilizing the protein complex during the molecular dynamics simulations (Fig. 2A). This stiffening effect can also be detected on the single elements of the complex as TF (Fig. 2B), FVIIa H-chain (Fig. 2C) and FVIIa L-chain (Fig. 2D). These suggestions are further supported by RainbowRMSD analysis (Fig. 2E, 2F, 2G, 2H).

**Figure 3:**
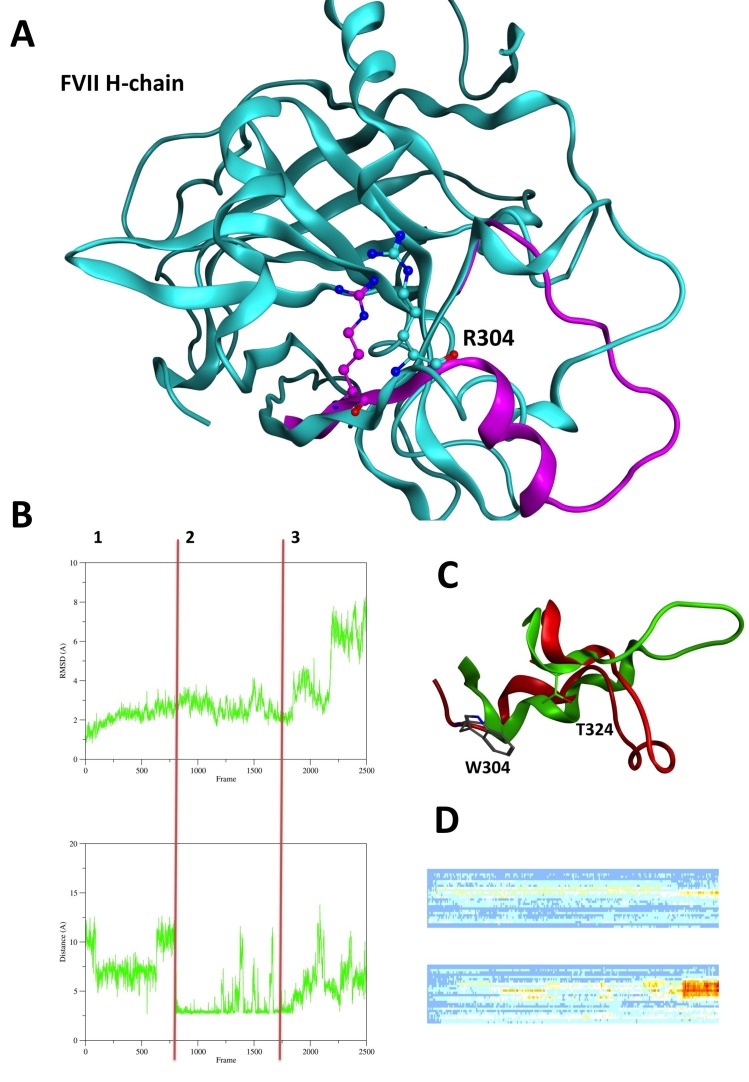
Heavy and light chain interactions with Tissue Factor. A, The illustration depicts the ribbon of the FVII WT zymogen H-chain (cyan) (PDB: 1JBU). The Val302-Met327 loop of FVIIa wt (in complex with TF, PDB: 1J9C) is represented in magenta ribbon. Arg304 is represented in cyan/magenta balls and sticks; B, The first graph depicts the RMSD over 100ns NVT 300K MD simulation of the loop Val302-Met327 of FVII “zymogen” Arg304Gln. The second graph shows the distance evolution of the polar interaction between Trp304 and Thr324. During phase 1 of the MD run the RMSD reaches the “plateau” and during phase 2 Trp304-Thr324 interaction is formed. During phase 3 this interaction is broken and an increase in RMSD values has been evidenced; C, The image illustrates the superimposition of the loop Val302-Met327 of the starting conformation (green) and of the conformation reached after 100ns NVT 300K MD simulation (red). Trp304 and Thr324 have been represented in sticks; D, The first picture illustrates the heatmap of the loop “Val302-Met327” RMSD overtime of FVII WT. The second illustration shows the heatmap of the loop “Val302-Met327” RMSD overtime of FVII Arg304.

RMSD overtime (Fig. 3B) and heatmaps (Fig. 3D) of Val302-Met327 loops of wt and Arg304Gln FVII shows major conformational changes of moiety when the Arg304Gln mutation occurs (Fig. 3A). These results are supported by distance analysis of salt-bridge interaction Arg304-Glu270 in FVII wt (Fig. 3B) and H-bond interaction Gln304-Thr324 (picture not shown). Arg304-Glu270 interaction is almost formed during the entire wt simulation run (except at 50ns and between 75 ns and 90 ns). On the contrary, Gln304-Thr324 is prevalently formed between 0 and 30ns of MD simulation. Arg304Gln mutation avoids the formation of a salt-bridge network, which influences the protein conformation of the loop Val302-Met327 permitting a conformational change not depicted for the wt simulation.

Arg304Trp mutation is able to influence the loop Val302-Met327 conformation, too.

The RMSD overtime analysis of the moiety (Fig. 3B) shows three phases of the MD simulation. In the first phase (0ns to 30ns), the RMSD trend reaches the plateau and it corresponds to the period of the simulation while there is the formation of a polar interaction of a polar interaction between Trp304 and Thr324 (Fig. 3C).

## DISCUSSION

The mutation Arg79Gln is located in the first epidermal growth factor section of the light chain. On the contrary, the Arg304Gln or Arg304Trp mutations are localized in the catalytic region of the heavy chain. The former is encoded by exon 4 whereas the latter are encoded by exon 8 (11).

The clotting patterns of these three conditions granting the potential small variations, stemming from the different settings, are similar. In all cases, FVII activity results low using rabbit brain reagents, intermediate with thromboplastins of human origin (human placenta or human recombinant) and near normal, or even normal, when ox brain thromboplastin is used. Furthermore, FVII antigen is normal in all cases (5, 8-10).

It is worth noting that a mutation in site 303, the Pro303Thr (33), does not show these thromboplastin dependent variations in FVII activity level in spite of a normal FVII antigen and in spite of the vicinity to the Arg304 involved both in FVII Padua and FVII Nagoya. This indicates the pivotal role of Arg. In fact, the substitution of an arginine removes a positively charged guanidium side chain (guanidium group) (34). Whenever an arginine is lost, regardless of its position, the interaction with tissue factor can be altered and this explains also the conformational changes. In fact, neither glutamine nor tryptophan has such guanidium group.

The analysis of crystal structures and the molecular dynamics studies suggest that all these three mutations cause a freezing in the protein conformation of FVIIa that could be interpreted as a decreased compliance of the FVII molecule in interacting with TF. The RMSD graphs depicted in Fig. 2, of the present molecular dynamics simulations, show higher values for the protein RMSD of the wt model compared to mutants. Particularly, the discrepancy with the wt is more evident for the heavy chain (Fig. 2C) compared to the light chain (Fig. 2D), indicating a preponderant role of the Arg304, which is contained in the catalytic domain of the heavy chain. RainbowRMSD (Fig. 2E, 2F, 2G, 2H) analysis support the results depicted in the RMSD graphs of Fig. 2.

The reduced FVII conformational flexibility of tested variants results in variable levels of activity according to the composition of the thromboplastin used. The mechanism of action of tissue factor binding is still not completely clarified (35). It has been postulated that the Arg79 first tethers FVII to TF and, after this event, Arg304 completes the binding and activation processes (36, 37). The effect of Arg304, besides binding, seems crucial for the full FX activation process (38).

Other studies (39) indicate that the role of EGF in tissue factor binding causes an alignment of tissue factor with the catalytic domain of activated FVII. Such alignment is responsible for optimal catalytic activity.

Factor VII defects due to mutation Arg79Gln (FVII Shinjo or Tondabayashi) and to Arg304Gln (FVII Padua) have a peculiar common feature, namely they play a role in tissue binding (5, 8-10). This is surprising because of the different location of the two mutations.

Interestingly, the same amino acid, Arg, is mutated with Gln in both cases (Factor VII Padua and FVII Shinjo or Tondabayashi) or with Trp (FVII Nagoya).

The FVII Arg304Trp (FVII Nagoya) mutation shows a coagulation profile identical to FVII Padua, confirming the pivotal role of Arg304 in tissue factor binding and activation. No homozygote Arg79Trp or with another substitute has been described so far, and therefore no comparison can be drawn.

The conformational studies here presented are in agreement with this interpretation. The molecular dynamics results of the RMSD analysis indicate that all these mutations influence the FVIIa: TF conformation in the same way: stabilizing the protein complex and conformation. The lack of the protein flexibility, depicted in the FVIIa: TF mutated models, but absent in the wt FVIIa could support the hypothesis of the formation of a defective FVIIa: TF complex, which fully justifies the variable FVII activity level obtained in the different assay systems.

The molecular dynamics results support the hypothesis that a more rigid conformation of the FVIIa: TF complex by Arg304Gln, Arg304Trp, Arg79Gln mutants negatively influence the cleavage of the subsequent factors in the coagulation cascade, namely FX.

Our computational studies on the FVII “Zymogen” support the hypothesis that both Arg304Gln and Arg304Trp act at the molecular level on the conformation of loop Val302-Met327, which is directly involved in the interaction with the TF during the process of FVII activation.

Finally, these studies indicate that crystal structure visual inspection and analysis coupled to large-scale molecular dynamics simulations may supply useful information in the evaluation of structure-function relationship between coagulation factors and the influence of mutations at molecular level (40, 41).
